# Genetic Diversity and Characteristics of *bla*_NDM_-Positive Plasmids in *Escherichia coli*

**DOI:** 10.3389/fmicb.2021.729952

**Published:** 2021-11-16

**Authors:** Zhiren Zhang, Hengzhao Guo, Xiaodong Li, Wenting Li, Guang Yang, Wenjun Ni, Meixiao Zhan, Ligong Lu, Zhenlin Zhang, Xiaobin Li, Zhiling Zhou

**Affiliations:** ^1^Zhuhai Precision Medical Center, Zhuhai People’s Hospital (Zhuhai Hospital Affiliated With Jinan University), Zhuhai, China; ^2^Department of Radiation Oncology, Zhuhai People’s Hospital (Zhuhai Hospital Affiliated With Jinan University), Zhuhai, China; ^3^Department of Spine and Osteology, Zhuhai People’s Hospital (Zhuhai Hospital Affiliated With Jinan University), Zhuhai, China; ^4^Department of Urology, Zhuhai People’s Hospital (Zhuhai Hospital Affiliated With Jinan University), Zhuhai, China; ^5^Zhuhai Interventional Medical Center, Zhuhai People’s Hospital (Zhuhai Hospital Affiliated With Jinan University), Zhuhai, China; ^6^Department of Clinical Laboratory, Zhuhai People’s Hospital (Zhuhai Hospital Affiliated With Jinan University), Zhuhai, China; ^7^Department of Pharmacy, Zhuhai People’s Hospital (Zhuhai Hospital Affiliated With Jinan University), Zhuhai, China

**Keywords:** *Escherichia coli*, New Delhi metallo-β-lactamase, plasmid, conjugative, mobilizable

## Abstract

New Delhi metallo-β-lactamases (NDMs), including at least 28 variants, are a rapidly emerging family of β-lactamases worldwide, with a variety of infections caused by NDM-positive strains usually associated with very poor prognosis and high mortality. NDMs are the most prevalent carbapenemases in *Escherichia coli* (*E. coli*) worldwide, especially in China. The vast majority of *bla*_NDM_ cases occur on plasmids, which play a vital role in the dissemination of *bla*_NDM_. To systematically explore the relationships between plasmids and *bla*_NDM_ genes in *E. coli* and obtain an overall picture of the conjugative and mobilizable *bla*_NDM_-positive plasmids, we analyzed the variants of *bla*_NDM_, replicon types, phylogenetic patterns, conjugative transfer modules, host STs, and geographical distributions of 114 *bla*_NDM_-positive plasmids, which were selected from 3786 plasmids from 1346 complete whole genomes of *E. coli* from the GenBank database. We also established links among the characteristics of *bla*_NDM_-positive plasmids in *E. coli*. Eight variants of *bla*_NDM_ were found among the 114 *bla*_NDM_-positive plasmids, with *bla*_NDM__–__5_ (74 *bla*_NDM__–__5_ genes in 73 plasmids), and *bla*_NDM__–__1_ (31 *bla*_NDM__–__1_ genes in 28 plasmids) being the most dominant. The variant *bla*_NDM__–__5_ was mainly carried by the IncX3 plasmids and IncF plasmids in *E. coli*, the former were mainly geographically distributed in East Asia (especially in China) and the United States, and the latter were widely distributed worldwide. IncC plasmids were observed to be the predominant carriers of *bla*_NDM__–__1_ genes in *E. coli*, which were mainly geographically distributed in the United States and China. Other *bla*_NDM__–__1_-carrying plasmids also included IncM2, IncN2, and IncHI1. Moreover, the overall picture of the conjugative and mobilizable *bla*_NDM_-positive plasmids in *E. coli* was described in our study. Our findings enhance our understanding of the genetic diversity and characteristics of *bla*_NDM_-positive plasmids in in *E. coli*.

## Introduction

New Delhi metallo-β-lactamase (NDM) is a metallo-β-lactamase that can hydrolyze almost all β-lactam antibiotics, including carbapenems (Nordmann et al., 2011). NDM-1 was first identified in a *Klebsiella pneumoniae* strain isolated from a Swedish patient who had been hospitalized in New Delhi, India in 2008 (Yong et al., 2009). So far, 28 variants of NDM have been reported (Farhat and Khan, 2020). A variety of infections caused by NDM-positive strains are reportedly associated with very poor prognosis and high mortality, especially in neonates and high-risk immunocompromised patients (Guducuoglu et al., 2018). NDM-positive strains have been found worldwide, with the highest prevalence in the Indian subcontinent, the Middle East, and the Balkans (Albiger et al., 2015; Wu and Feng, 2019). According to the Study for Monitoring Antimicrobial Resistance Trends (SMART) global surveillance program, *bla*_NDM_ is the third most common carbapenemase-encoding gene and accounts for 19.42% of carbapenemase positivity after *bla*_KPC_ (53.18%) and the *bla*_OXA__–__48_-like gene (20.09%) (Karlowsky et al., 2017). In China, the presence of *bla*_KPC_ (51.6%) and *bla*_NDM_ (35.7%) is responsible for phenotypic resistance in most carbapenem-resistant Enterobacteriaceae (CRE) strains (Han et al., 2020), according to data from the China Antimicrobial Surveillance Network (CHINET) in 2018. Furthermore, data from SMART and CHINET2018 demonstrated that NDM was the most prevalent carbapenemase in *E. coli*, especially in China; *bla*_NDM_ accounted for 93.0 and 97.2% of carbapenem-resistant *E. coli* isolates from adults and children, respectively (Han et al., 2020).

Antimicrobial resistance (AMR) in CRE isolates is frequently mediated by plasmid-borne genes, in addition to chromosomal determinants (Rozwandowicz et al., 2018). Plasmids remain important microbial components that mediate horizontal gene transfer (HGT) and play a vital role in the dissemination of AMR (Jiang et al., 2020). *bla*_NDM_ has been reported to be carried on plasmids with a variety of replicon types, most of which belong to limited replicon types (IncX3, IncFII, or IncC) (Wu and Feng, 2019). For CRE isolates, conjugative plasmids have been highlighted as important vehicles for the dissemination of AMR (Smillie et al., 2010; Ravi et al., 2018). The conjugative transfer regions of the conjugative plasmids typically consist of four modules: an origin of transfer (*oriT*) region, relaxase gene, type IV coupling protein (T4CP) gene, and gene cluster for the bacterial type IV secretion system (T4SS) apparatus (de la Cruz et al., 2010). In addition, mobilizable plasmids are also contributors to AMR, typically carrying the indispensable *oriT* sites and a limited number of *mob* genes for their own DNA processing in conjugation, which can be mobilized by conjugative elements (Ramsay and Firth, 2017). Currently, studies on the distribution of conjugative and mobilizable *bla*_NDM_-positive carbapenem-resistant plasmids in *E. coli* are rare. With the increase in the amount of whole-genome/plasmid sequencing data, there is a need for large-scale plasmid analysis of *bla*_NDM_-positive plasmids of *E. coli*.

In this study, we performed *in silico* typing and comparative analysis of *bla*_NDM_-positive plasmids of *E. coli* using the bacterial genome and plasmid sequences available in the NCBI database. We analyzed the geographical distribution of *bla*_NDM_-positive plasmids and compared the replicon types, conjugative transfer modules, and profiles of resistance determinants among *bla*_NDM_-positive plasmids of *E. coli*. This study provides important insights into the phylogeny and evolution of *bla*_NDM_-positive *E. coli* plasmids and further addresses their role in the acquisition and spread of resistance genes.

## Materials and Methods

For this study, the data collection and analysis are shown in Supplementary Figure 1.

### Plasmid Genomic Sequences

A total of 1346 complete whole genomes of *E. coli*, including the genomes marked by “Chromosome” and “Complete” in assembly level, were downloaded from the GenBank (Benson et al., 2018) Genome database.^1^ The download date was April 15, 2021. We extracted 3786 plasmid genomic sequences without duplicates (Supplementary Table 1) from the 1346 complete whole genomes of *E. coli.* In addition, a total of 35150 bacterial plasmid genomic sequences were downloaded from the NCBI RefSeq database (O’Leary et al., 2016),^2^ including 6054 plasmids from *E. coli* (Supplementary Table 2), with the download date as July 14, 2021. The genome data (FASTA DNA format) were downloaded in bulk into a DELL PowerEdge R930 server with a Linux-CentOS7 operating system, using two Bioperl modules (Bio:DB:GenBank and Bio:SeqIO). Perl v5.16.3 was installed in the Linux platform.

### Determination of *bla*_NDM_-Positive Plasmids of *E. coli*

The potential β-lactamase genes of plasmids in FASTA DNA format were determined using the ResFinder software version 4.1^3^ (Bortolaia et al., 2020) installed in our server, with a minimum coverage of 60%, minimum identity of 90%, and species of “*Escherichia coli.*” The term “*bla*_NDM_” was used to search in the “Resistance gene” list within the ResFinder results to determine the *bla*_NDM_-positive plasmids of *E. coli*.

### Replicon Sequence Analysis of the *bla*_NDM_-Positive Plasmids of *E. coli*

Plasmid replicon typing was performed using the PlasmidFinder software^4^ (Carattoli and Hasman, 2020). Selecting the database “Enterobacteriaceae,” the DNA files in FASTA format were analyzed in bulk using the PlasmidFinder software version 2.0.1 installed in the Linux platform, with the minimum coverage of 60% and minimum identity of 95%.

### Phylogenetic Analyses of the *bla*_NDM_-Positive Plasmids of *E. coli*

The files in GenBank format of the *bla*_NDM_-positive plasmids of *E. coli* were downloaded in bulk using the Bio:DB:GenBank and Bio:SeqIO modules. Files containing protein sequences were extracted from the files in GenBank format using the Bioperl/Bio:SeqIO module. Phylogenetic patterns based on the presence/absence of orthologous gene families of all *bla*_NDM_-positive plasmids of *E. coli* were analyzed in this study. A binary protein presence/absence matrix was created using OrthoFinder^5^ (Emms and Kelly, 2019) with DIAMOND for sequence similarity searches, and then a hierarchical cluster result was shown by iTOL^6^ (Letunic and Bork, 2016).

### Characterization of the Conjugative Modules of *bla*_NDM_-Positive Plasmids

The files in GenBank format of the *bla*_NDM_-positive plasmids of *E. coli* were analyzed in bulk using the software oriTfinder^7^ (Li et al., 2018) (local version) to determine the presence/absence of *oriT*s, relaxase genes, T4CP genes, and gene cluster for T4SS. Furthermore, the types of *oriT*s, relaxase genes, T4CP genes, and gene cluster for T4SS toward the plasmids were identified based on the exhibition of oriTDB database^8^ (Li et al., 2018).

### Multilocus Sequence Typing of *E. coli* Strains Bearing *bla*_NDM_-Positive Plasmids

The *bla*_NDM_-positive plasmid-matched host *E. coli* strains were collected, and their DNA fasta sequences were downloaded in bulk using the Bio:DB:GenBank and Bio:SeqIO modules. The MLST software (Larsen et al., 2012) version 2.0.4 was downloaded from the website^9^ and installed on the Linux platform. The genomes of *E. coli* strains were analyzed in bulk using MLST software. The “*Escherichia_coli#1*” dataset containing the seven housekeeping genes (*adk*, *fumC*, *gyrB*, *icd*, *mdh*, *purA*, and *recA*) (Wirth et al., 2006) was selected.

## Results

### General Characteristics of *bla*_NDM_-Positive Plasmids of *E. coli*

Using ResFinder, 1001 (26.4%) plasmids bearing β-lactamase genes were identified from the 3786 plasmids, which were included in the 1346 complete whole genomes of *E. coli*. Among the 1001 plasmids containing β-lactamase genes, 114 (11.6%) were further identified as *bla*_NDM_-positive plasmids, which were distributed in 113 strains of *E. coli*.

**FIGURE 1:**
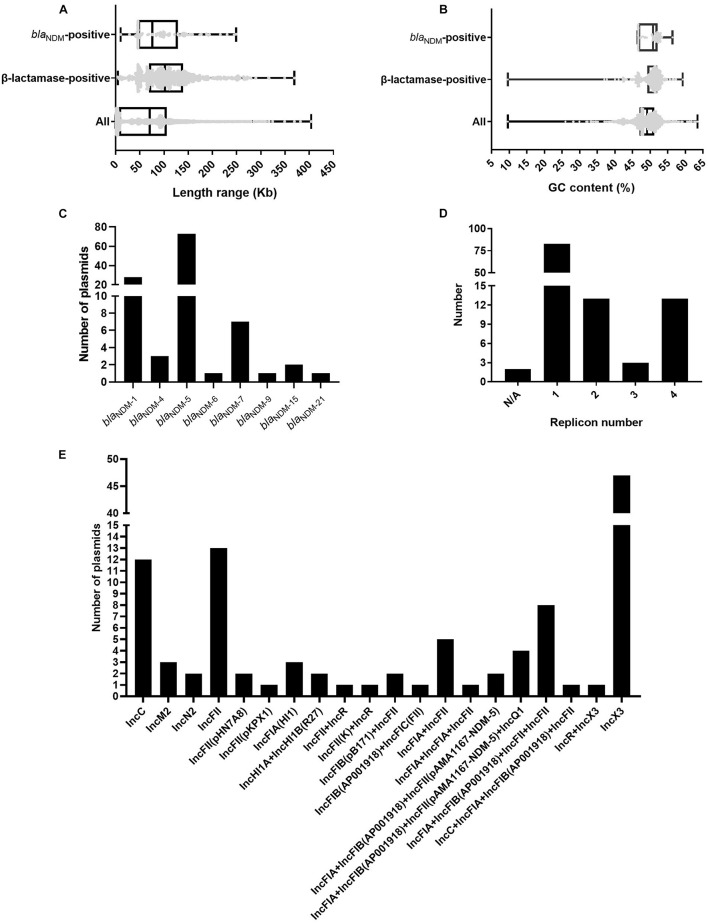
Characteristics of the 114 *bla*_NDM_-positive plasmids from 113 completely sequenced *E. coli* genomes. **(A)** Box plot of the length distribution of the 114 *bla*_NDM_-positive plasmids, the 1001 plasmids carrying β-lactamase genes, and all 3786 plasmids of *E. coli*. **(B)** Box plot of the GC content distribution of the 114 *bla*_NDM_-positive plasmids, the 1001 plasmids carrying β-lactamase genes, and all 3786 plasmids of *E. coli*. **(C)** Histogram of number of variants of *bla*_NDM_ genes among the 114 *bla*_NDM_-positive plasmids. **(D)** Histogram of number of replicons per plasmid for the 114 *bla*_NDM_-positive plasmids. **(E)** Histogram of number of combination modes of different replicons among the 114 *bla*_NDM_-positive plasmids.

We analyzed and compared the genome sizes of the *bla*_NDM_-positive plasmids, plasmids containing β-lactamase genes, and all 3786 plasmids of *E. coli.* Among the 113 fully sequenced *E. coli* strains, the genome sizes of 114 *bla*_NDM_-positive plasmids varied from 10.49 to 248.8 kb, with the 25% percentile, median, and 75% percentile were 46.16, 75.6, and 128.8 kb, respectively (Figure 1A). For the β-lactamase gene-positive plasmids and all 3786 plasmids of *E. coli*, their genome sizes varied greatly. Genome sizes of the former ranged from 4.49 to 369.3 kb and those of the latter ranged from 0.3 to 404.2 kb (Figure 1A).

We calculated the GC contents of the *bla*_NDM_-positive plasmids, plasmids containing β-lactamase genes, and all 3786 plasmids of *E. coli.* The GC content of the 114 *bla*_NDM_-positive plasmids ranged from 46.5 to 56.4%, with a median GC content of 50.8% (25% percentile = 46.7%; 75% percentile = 52.2%) (Figure 1B). For the plasmids containing β-lactamase genes and all 3786 plasmids of *E. coli*, the range of GC contents varied greatly. The GC content of the former ranged from 9.6 to 59.3%, and those of the latter ranged from 9.6 to 63.5% (Figure 1B).

### Variants of *bla*_NDM_ Genes in the *bla*_NDM_-Positive Plasmids of *E. coli*

Among the 114 *bla*_NDM_-positive plasmids, 124 *bla*_NDM_ genes belonging to eight kinds of variants of *bla*_NDM_, including *bla*_NDM__–__1_, *bla*_NDM__–__4_, *bla*_NDM__–__5_, *bla*_NDM__–__6_, *bla*_NDM__–__7_, *bla*_NDM__–__9_, *bla*_NDM__–__15_, and *bla*_NDM__–__21_, were identified. Among the eight variants of *bla*_NDM_, *bla*_NDM__–__5_ was found to be the most dominant variant (74 *bla*_NDM__–__5_ genes in 73 plasmids), followed by *bla*_NDM__–__1_ (31 *bla*_NDM__–__1_ genes in 28 plasmids), and *bla*_NDM__–__7_ (7 *bla*_NDM__–__7_ genes in seven plasmids) (Figure 1C).

### Replicon Types of Plasmids Carrying *bla*_NDM_ of *E. coli*

Replicon typing of the 114 *bla*_NDM_-positive plasmids was performed using PlasmidFinder. Among the 114 plasmids, 112 successfully identified their replicon types, including 83 single-replicon plasmids and 29 multi-replicon plasmids (13 plasmids with two replicons, 3 plasmids with three replicons, and 13 plasmids with four replicons) (Figure 1D). For the 83 single-replicon plasmids, plasmids with an IncX3 replicon were found to be the most dominant single-replicon plasmids (47 plasmids), followed by plasmids with an IncFII replicon (13 plasmids) and those with an IncC replicon (12 plasmids) (Figure 1E). Interestingly, the multi-replicon plasmids were mainly classified into IncF plasmids (Figure 1E). In summary, all 114 *bla*_NDM_-positive plasmids were mainly classified into IncX3, IncF, and IncC plasmids (Supplementary Figure 2).

### Genetic Diversity of the *bla*_NDM_-Positive Plasmids of *E. coli*

**FIGURE 2:**
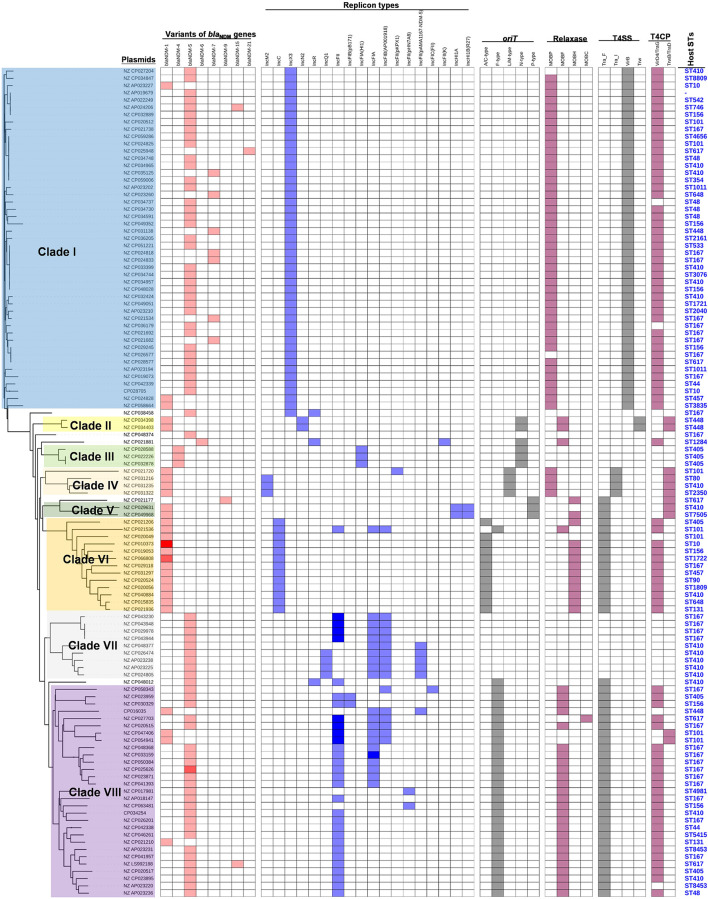
Details of variants of *bla*_NDM_ genes, replicon types of plasmids, and the conjugative transfer modules of the 114 *bla*_NDM_-positive plasmids in *E. coli*. The five categories of information present in this figure include the phylogenetic tree of 114 *bla*_NDM_-positive plasmids, variants of *bla*_NDM_ genes, replicon types, conjugative transfer modules, and STs of host strains. The gradient of color of each heatmap (variants of *bla*_NDM_ genes, replicon types, phylogenetic patterns, conjugative transfer modules including *oriT*, relaxase, T4CP, and T4SS) represents the variable numbers of genes or gene clusters.

To obtain a comprehensive overview of *bla*_NDM_-positive plasmids, we constructed phylogenetic trees of all 114 *bla*_NDM_-positive plasmids (Figure 2). Based on the phylogenetic patterns of plasmids, combined with the plasmid types and conjugative transfer modules, 109 of the 114 *bla*_NDM_-positive plasmids were classified into eight main clades (Clade I—Clade VIII), representing eight representative plasmid patterns carrying *bla*_NDM_ genes in *E. coli*. We also investigated the geographical distribution of the eight clades from *bla*_NDM_-positive *E. coli* plasmids.

**FIGURE 3:**
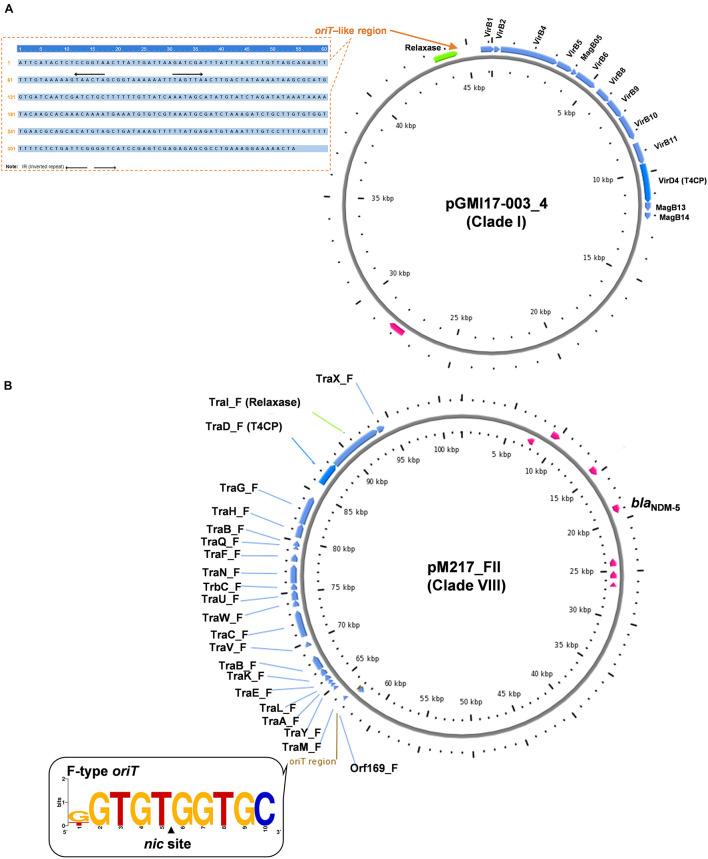
Conjugative transfer modules including *oriT*, relaxase, T4CP, and T4SS of the representative plasmids from Clade I **(A)** and Clade VIII **(B)**, respectively.

**FIGURE 4:**
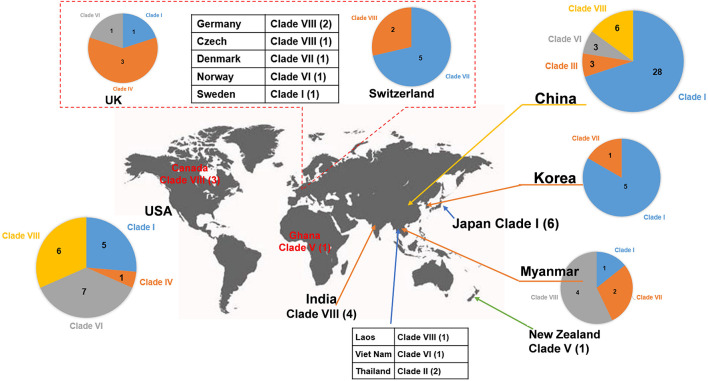
Worldwide distribution of *bla*_NDM_-positive plasmids of *E. coli*. The geographical distribution of the eight clades (Clade I–Clade VIII) from the *bla*_NDM_-positive plasmids of *E. coli* was calculated and displayed by pie chart.

Clade I: A total of 47 plasmids were identified in the Clade I cluster, accounting for approximately 41.2% of all 114 *bla*_NDM_-positive plasmids, which is the clade with the largest number among all the eights clades (Figure 2). Most (76.6%) of the plasmids classified into Clade I carried *bla*_NDM__–__5_ gene. All plasmids below Clade I were single-replicon plasmids with an IncX3 replicon, and most were 46-kb plasmids. For the conjugative transfer modules, almost all the plasmids belonging to Clade I carried relaxases of the MOB_P_ family and T4CPs of the VirD4/TraG subfamily. All 47 Clade I plasmids carrying *bla*_NDM_ were found to contain VirB-type T4SS gene clusters (Figure 3A). The current version of oriTfinder could not identify the *oriT* sites of the Clade I plasmids, while 354-bp intergenic sequences flanking the relaxase genes were *oriT*–like regions, with the inverted repeat (IR) sequence (TAACTA.TAGTTA) (Figures 2, 3A). The STs of *E. coli* host strains containing all Clade I plasmids were distributed in ST167, ST410, ST48, etc. For the clade with the largest number, Clade I, its plasmids were mainly distributed in East Asia (especially in China) and the United States (Figure 4). Most of the Clade I plasmids were the human origin, some were animal origin (mainly in China) and environment origin (both in Japan and China) (Supplementary Table 3).

Clade II: Two *bla*_NDM__–__1_-positive IncN2 plasmids of *E. coli* ST448 were clustered into Clade II (Figure 2). They both carried the N-type *oriT*s, relaxases of MOB_*F*_ family, T4CPs of TrwB/TraD subfamily, and Trw type of T4SS gene clusters (Figure 2 and Supplementary Figure 3). Clade II, containing two *bla*_NDM__–__1_-positive IncN2 plasmids, was found to be distributed only in Thailand (human origin) (Figure 4 and Supplementary Table 3).

Clade III: Three *bla*_NDM__–__4_-positive IncF plasmids with IncFIA(HI1) replicon of *E. coli* ST405 were clustered into Clade III (Figure 2). They were all found to carry only one conjugative transfer module: N-type *oriT*s, but no relaxases, T4CPs, or T4SS gene clusters were found, indicating that they should be mobilizable plasmids (Supplementary Figure 4). All three members of Clade III, inferred as mobilizable plasmids, were only found to be geographically distributed in China (all human origin) (Figure 4 and Supplementary Table 3).

Clade IV: Four *bla*_NDM__–__1_-positive single-replicon plasmids were classified into Clade IV, including three IncM2 plasmids and one IncFII(pKPX1) plasmid (Figure 2). For the conjugative transfer modules of the four Clade IV plasmids, they all carry the L/M-type *oriT*s, relaxases of MOB_P_ family, T4CPs of TrwB/TraD subfamily, and Tra_I type of T4SS gene clusters (Figure 2 and Supplementary Figure 5). *E. coli* host strains containing all four Clade IV plasmids were distributed into four different STs. Members of Clade IV were mainly geographically distributed in the United Kingdom (three plasmids, all human origin) (Figure 4 and Supplementary Table 3).

Clade V: Two *bla*_NDM__–__1_-positive multi-replicon plasmids, both containing IncHI1A and IncHI1B(R27) replicons, were grouped into the clade V cluster (Figure 2). They both carried the P-type *oriT*s, T4CPs of the TrwB/TraD subfamily, and Tra_F type of T4SS gene clusters (Figure 2 and Supplementary Figure 6). One plasmid of *E. coli* ST7505 was found to carry a relaxase of the MOB_H_ family; the other plasmid of *E. coli* ST410 was not able to identify the relaxase gene in the genome of the plasmid. The two members of Clade V were geographically distributed in Ghana and New Zealand, respectively (both human origin) (Figure 4 and Supplementary Table 3).

**FIGURE 5:**
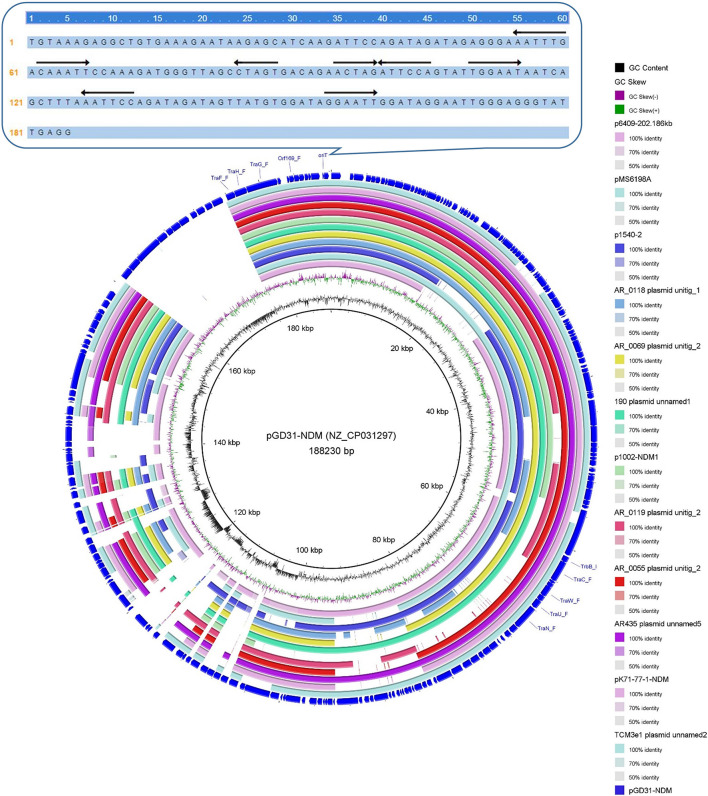
Conjugative transfer modules of 13 *bla*_NDM__–__1_-positive IncC plasmids grouped into the clade VI and the sequence logos of the flanking conserved regions of the *nic* sites of A/C-type *oriT* regions.

Clade VI: All 13 plasmids grouped into the clade VI cluster of the phylogenetic tree were found to carry *bla*_NDM__–__1_ gene (Figure 2). All plasmids belonging to Clade VI were identified as IncC plasmids, including 12 single-replicon plasmids with IncC replicon and one multi-replicon plasmid with four replicons (IncC, IncFIA, IncFIB(AP001918), and IncFII). For the conjugative transfer modules, most of the plasmids belonging to Clade VI carry the A/C-type *oriT*s, relaxases of MOB_H_ family and T4CPs of VirD4/TraG subfamily. All 13 clade VI plasmids carrying *bla*_NDM__–__1_ were found to have Tra_F type T4SS gene clusters (Figures 2, 5). No prevalent STs of *E. coli* host strains containing all Clade VI plasmids were found. The members of Clade VI were mainly geographically distributed in the United States and China (human origin) (Figure 4 and Supplementary Table 3).

Clade VII: Nine *bla*_NDM__–__5_-positive IncF plasmids were classified into clade VII (Figure 2). All the plasmids belonging to Clade VII were found to contain both replicon IncFIA and replicon IncFIB(AP001918), including two main kinds of replication combination modes: four plasmids with replicons IncFIA + IncFIB(AP001918) + IncFII + IncFII and four plasmids with replicons IncFIA + IncFIB(AP001918) + IncFII(pAMA1167-NDM-5) + IncQ1. Moreover, no conjugative transfer modules were found in the nine plasmids belonging to Clade VII, indicating that the nine plasmids should be non-transferable plasmids. The STs of *E. coli* host strains containing Clade VII plasmids were distributed into ST167 and ST410. The members of clade VII were mainly geographically distributed in Switzerland (Figure 4). In addition, clade VII members were also sporadically discovered in Myanmar, Korea, and Denmark. Clade VII plasmids included the human and animal origin (Supplementary Table 3).

Clade VIII: A total of 29 plasmids were identified in the Clade VIII cluster, accounting for approximately 25.4% of all 114 *bla*_NDM_-positive plasmids, which is the clade with the second largest number among all eights clades (Figure 2). Most (86.2%) of the plasmids grouped into clade VIII were found to carry *bla*_NDM__–__5_ gene. All plasmids below Clade VIII were IncF plasmids, with replicon IncFII as the most common replicons. For the conjugative transfer modules, all the plasmids belonging to Clade VIII carry the F-type *oriT*s and Tra_F type of T4SS gene clusters (Figures 2, 3B). Almost all the plasmids of Clade VIII had relaxases of the MOB_F_ family and T4CPs of the VirD4/TraG subfamily. The STs of *E. coli* host strains containing all Clade VIII plasmids were distributed in ST167, ST101, ST44, ST410 etc. For the clade with the second largest number, Clade VIII, its members were widely distributed all over the world, including East Asia, India, the United States, and some European countries (e.g., Germany, Switzerland, and the Czech Republic) (Figure 4). Most of the Clade VIII plasmids were the human origin, few were animal and environment origin (Supplementary Table 3).

We also analyzed the 6054 plasmids of *E. coli* downloaded from the NCBI RefSeq database, both with and without host strains. The results indicated that 301 *bla*_NDM_-positive plasmids were identified from the 6054 plasmids of *E. coli* (Supplementary Figure 7). We explored the distribution of the above eight representative plasmid patterns carrying *bla*_NDM_ genes of *E. coli* in the 301 *bla*_NDM_-positive plasmids. For the *bla*_NDM__–__5_ gene, it was mainly carried by the IncX3 plasmids (Clade I pattern) and IncF plasmids (Clade VII and Clade VIII patterns), consistent with the results based on the 114 *bla*_NDM_-positive plasmids with hosts. The IncN plasmids (including IncN2 and IncN replicons) were found to mainly carry the *bla*_NDM__–__1_ gene (Clade II pattern) in the 301 *bla*_NDM_-positive plasmids, consistent with the results from the 114 *bla*_NDM_-positive plasmids with hosts. The *bla*_NDM__–__4_ gene was carried sporadically by IncF, IncX, and IncR plasmids, not limited to the Clade III pattern from the 114 *bla*_NDM_-positive plasmids with hosts. The IncM2 plasmids were found to carry the *bla*_NDM__–__1_ gene (Clade IV pattern) in the 301 *bla*_NDM_-positive plasmids, consistent with the results of *bla*_NDM_-positive plasmids with hosts. For the IncHI1 and IncHI2 from the 301 *bla*_NDM_-positive plasmids, they were found to carry not only the *bla*_NDM__–__1_ (Clade V pattern) but also the *bla*_NDM__–__5_ gene. The IncC plasmids were found to carry the *bla*_NDM__–__1_ gene (Clade VI pattern) in the 301 *bla*_NDM_-positive plasmids, consistent with the conclusion from *bla*_NDM_-positive plasmids with hosts.

## Discussion

NDM carbapenemases are a rapidly emerging and troublesome family of β-lactamases (Pérez-Vázquez et al., 2019; Sugawara et al., 2019; Han et al., 2020). To explore the relationships among plasmids, *bla*_NDM_ genes, and hosts in *E. coli*, we systematically analyzed the profiles of resistance determinants, replicon typing, and comparative analysis of 3786 plasmids from 1346 complete whole genomes of *E. coli* from the GenBank database. Overall, 114 *bla*_NDM_-positive plasmids from 113 *E. coli* strains were identified.

Variants of *bla*_NDM_ included in the 114 *bla*_NDM_-positive plasmids in our study were classified into eight types. The *bla*_NDM__–__5_-carrying plasmids were the most common *bla*_NDM_-positive plasmids and accounted for 64.0% of the 114 *bla*_NDM_-positive plasmids, followed by *bla*_NDM__–__1_-positive plasmids (24.6%) and *bla*_NDM__–__7_-positive plasmids (6.1%). *bla*_NDM__–__1_ was first identified on a 180-kb plasmid of *K. pneumoniae* strain 05-506 and on a 140-kb plasmid carried by *E. coli* strain NF-NDM-1, both isolated from a Swedish patient who had been hospitalized in New Delhi, India, in 2008 (Yong et al., 2009). The variant NDM-5 was first detected in a strain of *E. coli* EC405 belonging to ST648, isolated from a 41-year-old patient in the United Kingdom with a history of travel to the Indian subcontinent, and *bla*_NDM__–__5_ was localized to an IncF plasmid with a length > 100 kb (Hornsey et al., 2011). The variant NDM-7 was first detected in a strain of *E. coli* ST599, isolated from the rectum, throat, and infected wounds of a Yemeni patient admitted to the Frankfurt University Hospital of Germany, and *bla*_NDM__–__7_ was localized on a self-transferable IncX3 plasmid of 60 kb (Göttig et al., 2013).

Among the 114 *bla*_NDM_-positive plasmids in *E. coli*, 112 were successfully identified by their replicon types, and mainly classified into IncX3, IncF, and IncC plasmids. Our results also indicated that the 112 *bla*_NDM_-positive plasmids contained 83 single-replicon plasmids and 29 multi-replicon plasmids.

IncX3 plasmids have been reported to carry various carbapenemase genes in CRE worldwide (Mouftah et al., 2019). Herein, our work indicated that the IncX3 plasmids were the most prevalent single-replicon plasmids among the 114 *bla*_NDM_-positive plasmids in *E. coli*, which were observed to be the predominant carriers of *bla*_NDM__–__5_ genes, distributed in Clade I of the phylogenetic profiles constructed by the 114 *bla*_NDM_-positive plasmids of *E. coli*. Common types of *E. coli* strains containing *bla*_NDM__–__5_-positive IncX3 plasmids were ST167, ST410, and ST48, located in East Asia (especially China) and the United States.

In our study, multi-replicon IncF plasmids, especially the plasmids with IncFII replicon, were another common carrier of *bla*_NDM__–__5_ genes, which were distributed in Clade VII and Clade VIII of the phylogenetic profiles of the 114 *bla*_NDM_-positive plasmids of *E. coli*, members of the former were identified as the non-transferable plasmids and those of the latter were identified as the conjugative plasmids. The IncF plasmids, widely distributed in Enterobacteriaceae, are known as conjugative plasmids that contribute to the carriage and spread of AMR genes (Carattoli, 2011), similar to Clade VIII of the phylogenetic profiles of the 114 *bla*_NDM_-positive plasmids of *E. coli* in our own study. However, in our study, we also found nine *bla*_NDM__–__5_-positive IncF plasmids without any classical conjugative transfer modules, classified into Clade VII of the phylogenetic patterns of the 114 *bla*_NDM_-positive plasmids of *E. coli*, which were identified as non-transferable plasmids. The nine non-transferable IncF plasmids of Clade VII were distributed in the *E. coli* strains of ST167 (four plasmids) and ST410 (five plasmids), mainly located in Switzerland. The *bla*_NDM_-positive IncF plasmids in *E. coli* grouped into Clade VIII, which were the classical conjugative plasmids, mainly distributed in the *E. coli* strains of ST167 and geographically distributed worldwide (East Asia, India, the United States, and some European countries).

IncC plasmids, almost all single-replicon plasmids, were observed to be the predominant carriers of *bla*_NDM__–__1_ genes in *E. coli*, which were grouped into Clade VI of the phylogenetic profiles constructed by the 114 *bla*_NDM_-positive plasmids of *E. coli* in this study. *E. coli* strains containing *bla*_NDM__–__1_-positive IncC plasmids belonged to a variety of STs, and no predominant STs were found, which were mainly geographically distributed in the United States and China. Other types of *bla*_NDM__–__1_-carrying plasmids included IncM2, IncN2, IncHI1, IncX3, and IncF. The large, broad host range IncC plasmids are important contributors to the spread of key antibiotic resistance genes, and over 200 complete sequences of IncC plasmids have been reported (Ambrose et al., 2018).

Bacterial mobile genetic elements, such as conjugative plasmids and transposons, have been highlighted as important vehicles for the dissemination of AMR, which play a central role in facilitating horizontal genetic exchange and therefore promoting the acquisition and spread of resistance genes (Partridge et al., 2018; Jiang et al., 2020). Currently, the genetic context of *bla*_NDM_ is mainly focused on the insertion sequences and transposons, for example, IS*Aba125*, IS*26*, and the composite transposon Tn*125* (Yong et al., 2009; Göttig et al., 2013; Partridge et al., 2018; Wu and Feng, 2019). In fact, conjugation is a dominant mechanism of HGT, and bacterial genome comparisons highlight conjugative and mobilizable elements as vehicles for dissemination of pathogenesis and AMR determinants (Ramsay and Firth, 2017). Reports on the distribution of various conjugative and mobilizable *bla*_NDM_-positive carbapenem-resistant plasmids in *E. coli* and their conjugative transfer modules are currently scarce. Herein, we performed a comprehensive analysis and comparison of the conjugative transfer modules located on the 114 *bla*_NDM_-positive plasmids using the software oriTfinder (Li et al., 2018), the database oriTDB (Li et al., 2018) and the database SecReT4 (Bi et al., 2013). The oriTDB database recorded nine types of plasmid-borne *oriT*^10^ (Li et al., 2018). In our study, five types of *oriT* regions were identified, including A/C-type *oriT*s in conjugative IncC plasmids carrying *bla*_NDM__–__1_ genes, F-type *oriT*s in conjugative IncF plasmids bearing *bla*_NDM__–__5_ genes, L/M-type *oriT*s in mostly *bla*_NDM__–__1_-positive conjugative IncM2 plasmids, N-type *oriT*s in both *bla*_NDM__–__1_-positive conjugative IncN2 plasmids and *bla*_NDM__–__4_-positive mobilizable plasmids with IncFIA(HI1) replicon, as well as P-type *oriT*s in *bla*_NDM__–__1_-positive conjugative IncHI1 plasmids. The oriTDB database recorded eight main relaxase families^11^ (Li et al., 2018), and four relaxase families were found in our study. Relaxases belonging to the MOB_P_ family were found in conjugative IncX3 plasmids and most of the conjugative IncM2 plasmids; Relaxases of MOB_F_ family were found in conjugative IncF plasmids and IncN2 conjugative plasmids; MOB_F_ relaxases were mostly found in conjugative IncC plasmids; the only one relaxase belonging to MOB_C_ was found in one conjugative IncF plasmid. The oriTDB database recorded two main subfamilies of T4CPs^12^ (Li et al., 2018). In our study, most T4CPs of conjugative IncX3, IncC, and IncF plasmids belong to the VirD4/TraG subfamily, while the T4CPs of conjugative IncN2, IncM2, and IncHI1 plasmids belong to the TrwB/TraD subfamily. The database SecReT4 collected the five main types of T4SS gene clusters, including 18 kinds of systems^13^ (Bi et al., 2013). Our study demonstrated that four kinds of T4SS gene clusters were found in the *bla*_NDM_-positive plasmids of *E. coli*, including the Tra_F-type of T4SS distributed in the conjugative IncF, IncC, and IncHI1 plasmids, VirB-type T4SS distributed in the conjugative IncX3 plasmids, Tra_I-type of T4SS mostly in conjugative IncM2 plasmids, Trw-type of T4SS in conjugative IncN2 plasmids.

## Conclusion

In this study, we analyzed the variants of *bla*_NDM_, replicon types, phylogenetic patterns, conjugative transfer modules, host STs, and geographical distributions of the 114 *bla*_NDM_-positive plasmids, from 3786 plasmids within 1346 complete whole genomes of *E. coli* from the GenBank database. Eight variants of *bla*_NDM_ were found among the 114 *bla*_NDM_-positive plasmids, with *bla*_NDM__–__5_ and *bla*_NDM__–__1_ as the most dominant. The variant *bla*_NDM__–__5_ was mainly carried by the IncX3 plasmids and IncF plasmids in *E. coli*, the former were mainly geographically distributed in East Asia (especially in China) and the United States, and the latter were widely distributed all over the world, including East Asia, Southeast Asia, India, the United States, and some European countries. IncC plasmids were observed to be the predominant carriers of *bla*_NDM__–__1_ genes in *E. coli*, which were mainly geographically distributed in the United States and China. Other *bla*_NDM__–__1_-carrying plasmids also included IncM2 (mainly geographically distributed in the United Kingdom), IncN2 (distributed in Thailand), and IncHI1 (in Ghana and New Zealand). In addition, the overall picture of the conjugative and mobilizable *bla*_NDM_-positive plasmids in *E. coli* was described in our study. The eight representative plasmid patterns carrying *bla*_NDM_ genes of *E. coli* was also validated with a larger data set (6054 plasmids of *E. coli* downloaded from the NCBI RefSeq database). This study provides important insights into the phylogeny and evolution of *bla*_NDM_-positive *E. coli* plasmids and further addresses their role in the acquisition and spread of resistance genes. However, the genetic diversity and characteristics of *bla*_NDM_ -positive plasmids in other Enterobacteriaceae species need further study in the future.

## Data Availability Statement

The original contributions presented in the study are included in the article/Supplementary Material, further inquiries can be directed to the corresponding author/s.

## Author Contributions

ZLZ and ZLZ conceived the project. ZRZ and XBL analyzed all the data and wrote the manuscript. WL, GY, and WN performed data acquisition. MZ and LL provided some suggestions for manuscript writing. HG and XDL reviewed and edited the manuscript. All authors read and approved the final manuscript.

## Conflict of Interest

The authors declare that the research was conducted in the absence of any commercial or financial relationships that could be construed as a potential conflict of interest.

## Publisher’s Note

All claims expressed in this article are solely those of the authors and do not necessarily represent those of their affiliated organizations, or those of the publisher, the editors and the reviewers. Any product that may be evaluated in this article, or claim that may be made by its manufacturer, is not guaranteed or endorsed by the publisher.

## Footnotes

1
https://www.ncbi.nlm.nih.gov/genome/browse/#!/prokaryotes/167/

2
https://ftp.ncbi.nih.gov/refseq/release/plasmid/

3
https://cge.cbs.dtu.dk/services/ResFinder/

4
https://cge.cbs.dtu.dk/services/PlasmidFinder/

5
http://www.stevekellylab.com/software/orthofinder

6
https://itol.embl.de/

7
https://bioinfo-mml.sjtu.edu.cn/oriTfinder/

8
https://bioinfo-mml.sjtu.edu.cn/oriTDB/index.php

9
https://cge.cbs.dtu.dk/services/MLST/

10
https://bioinfo-mml.sjtu.edu.cn/oriTDB/browse_oriT_type_p.php

11
https://bioinfo-mml.sjtu.edu.cn/oriTDB/browse_relaxase.php

12
https://bioinfo-mml.sjtu.edu.cn/oriTDB/browse_t4cp.php

13
https://db-mml.sjtu.edu.cn/SecReT4/browse_type.php
