# Supplementary material for: Genetic Diversity and Characteristics of blaNDM-Positive Plasmids in Escherichia coli
Source: Front Microbiol. 2021 Nov 16;12:729952. doi: 10.3389/fmicb.2021.729952 (PMC8636099; doi:10.3389/fmicb.2021.729952)
Supplement: Supplementary file 1 [file Data_Sheet_1.docx]

**Genetic diversity and characteristics of *bla*_NDM_-positive plasmids** **in *Escherichia coli***

**Figure legends:**

**Figure S1**. Flow diagram of collecting data and analyzing data in this study.

**Figure S2**. An overview of the oriTfinder-facilitated detection of putative *oriT* sites and three other conjugal modules among the 114 *bla*_NDM_-positive plasmids of *E. coli*. The phylogenetic patterns based on the presence/absence of orthologous gene families of all 114 *bla*_NDM_-positive plasmids under analysis. A binary gene presence/absence matrix was created using OrthoFinder and a hierarchical cluster result was visualized by iTOL. The plasmids belonging to the reported incompatibility groups (IncX3, IncF, and IncC) are marked in different colors. The four color circles denote the regions (or genes) present in a given plasmid, including *oriT*, relaxase, T4SS, and T4CP from the inside out. The gradient of color of each circle represents the variable numbers of genes or gene clusters.

**Figure S3**. Conjugative transfer modules of two *bla*_NDM-1_-positive IncN2 plasmids clustered into Clade II (A) and the sequence logos of the flanking conserved regions of the *nic* sites of N-type *oriT* regions (B).

**Figure S4**. Three *bla*_NDM-4_-positive IncF plasmids with IncFIA(HI1) replicon of *E. coli* ST405 clustered into Clade III and the sequence logos of the flanking conserved regions of the *nic* sites of N-type *oriT* regions.

**Figure S5**. Conjugative transfer modules of four *bla*_NDM-1_-positive plasmids clustered into Clade IV (A) and the sequence logos of the flanking conserved regions of the *nic* sites of L/M-type *oriT* regions (B).

**Figure S6**. Conjugative transfer modules of two *bla*_NDM-1_-positive IncHI1 plasmids clustered into Clade V (A) and the sequence logos of the flanking conserved regions of the *nic* sites of P-type *oriT* regions (B).

**Figure S7**. Details of variants of *bla*_NDM_ genes, replicon types of plasmids, and the conjugative transfer modules of the 301 *bla*_NDM_-positive plasmids in *E. coli*. The four categories of information present in this figure include the phylogenetic tree of 301 *bla*_NDM_-positive plasmids, variants of *bla*_NDM_ genes, replicon types, and conjugative transfer modules. The gradient of color of each heatmap (variants of *bla*_NDM_ genes, replicon types, phylogenetic patterns, conjugative transfer modules) represents the variable numbers of genes or gene clusters.

**Table legends：**

**Table S1.** Information of 3786 plasmids from 1346 complete whole genomes of *E. coli* included in this study.

**Table S2.** Information of 6054 plasmids of *E. coli* downloaded from the NCBI RefSeq database.

**Table S3.** Clinical data of the 113 strains of *E. coli*.


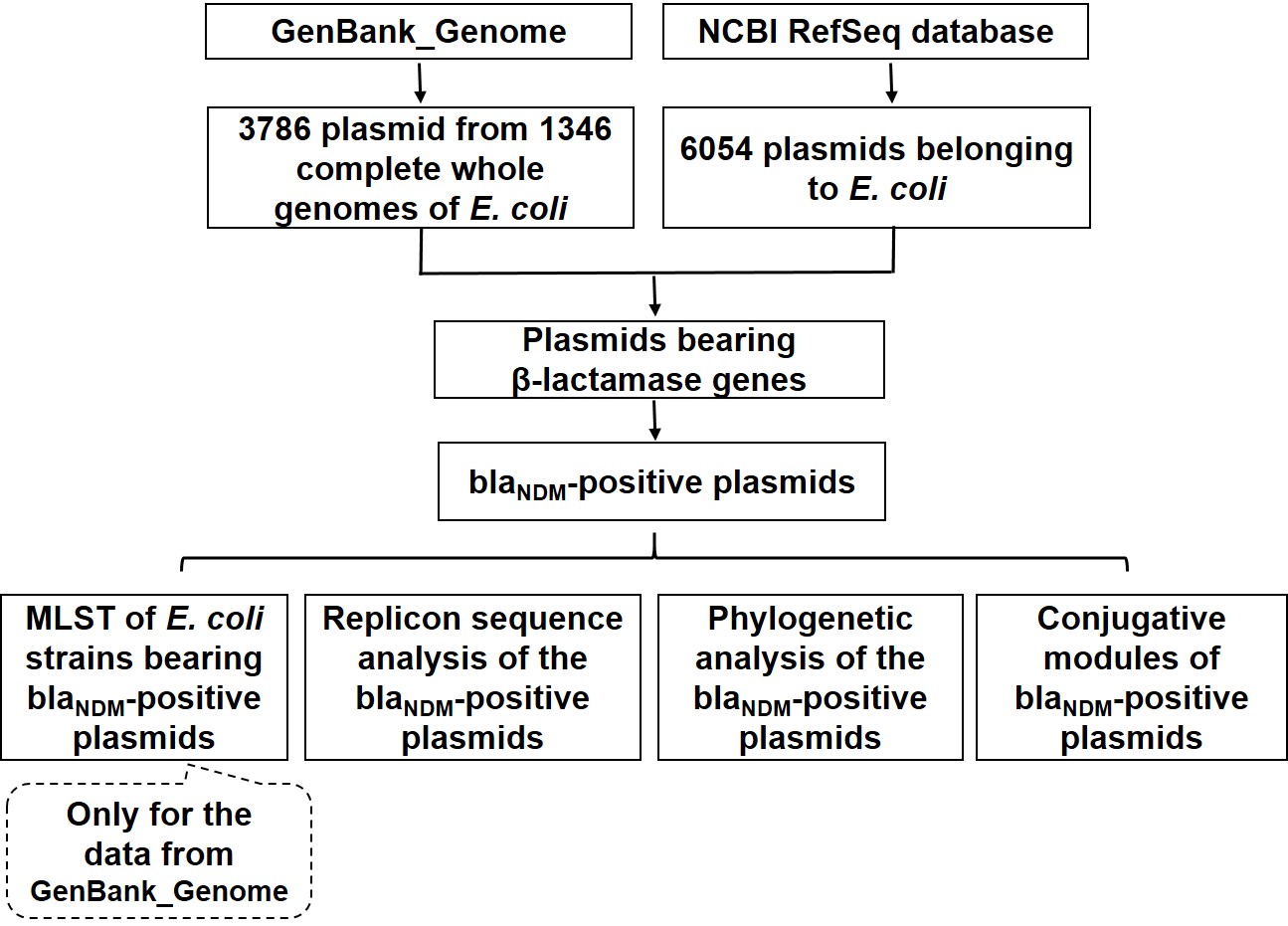


Figure S1


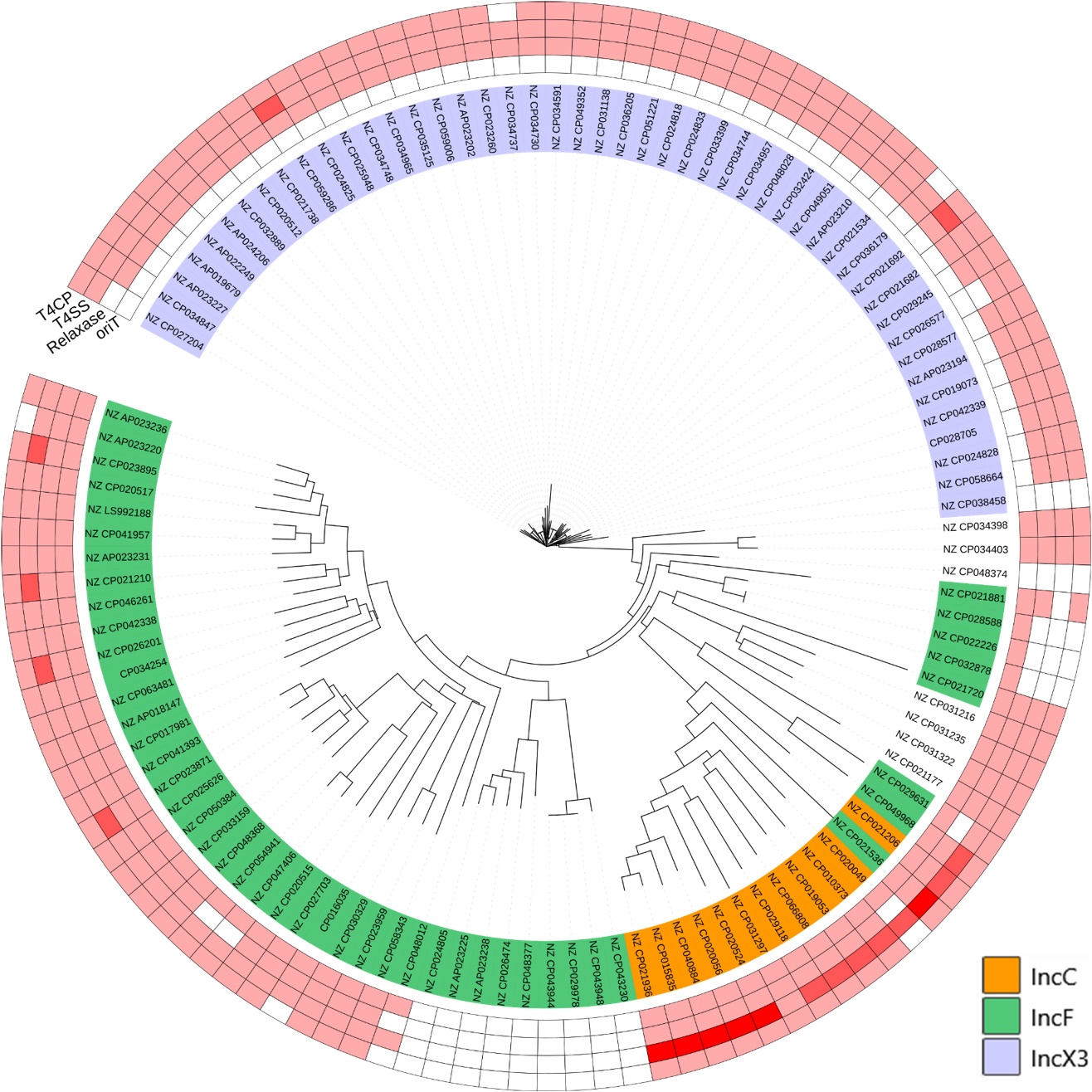


Figure S2


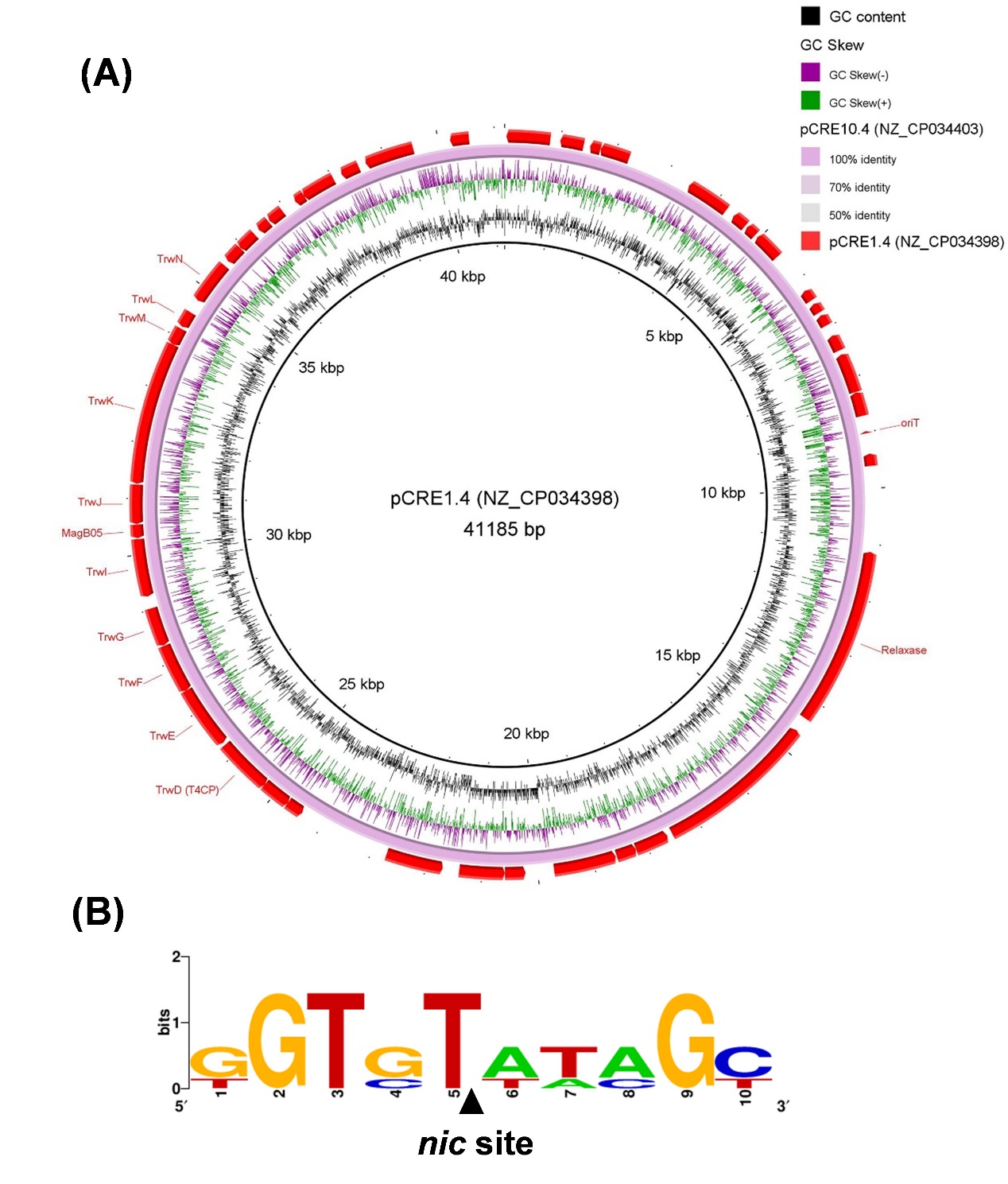


Figure S3


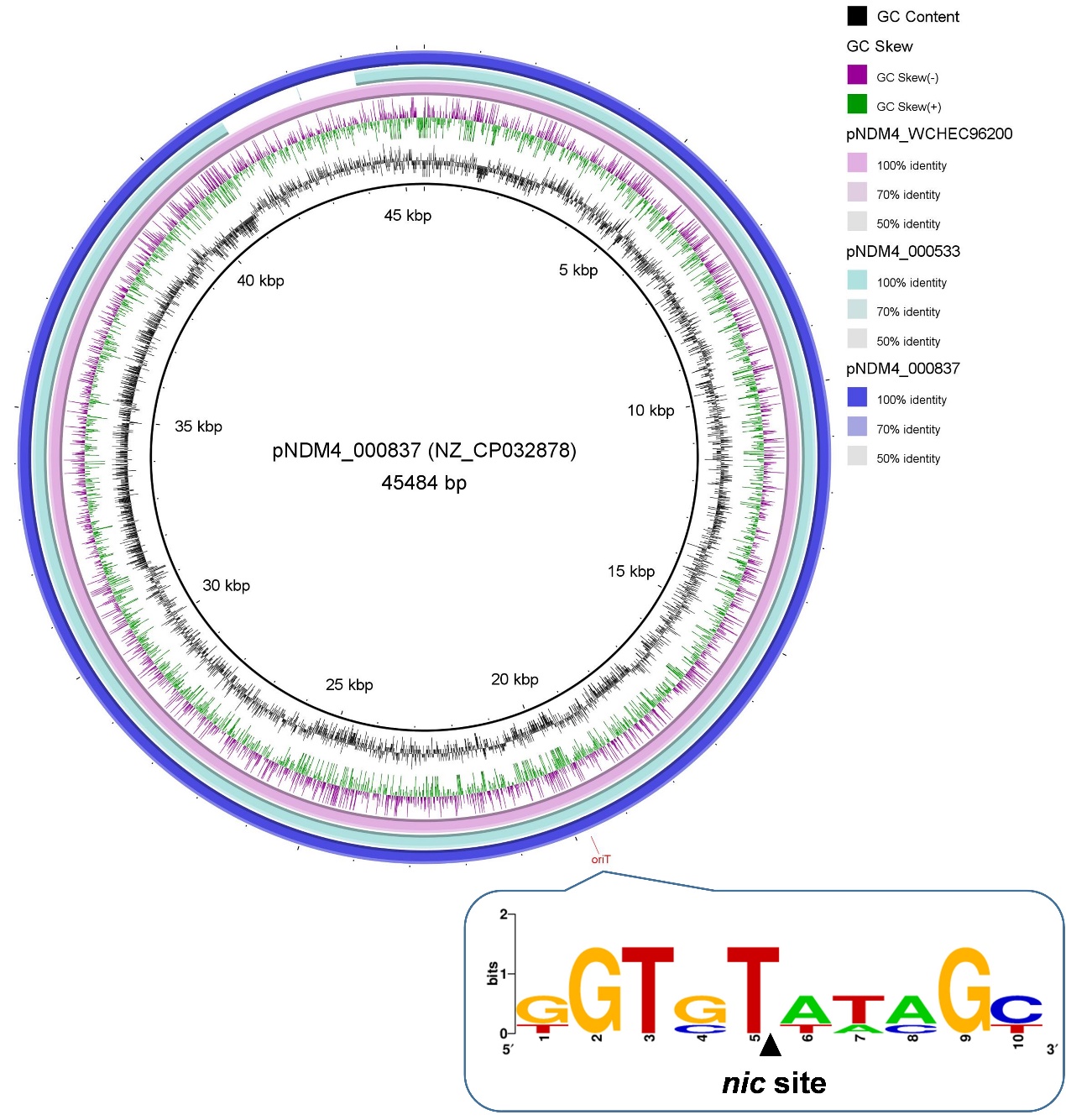


Figure S4


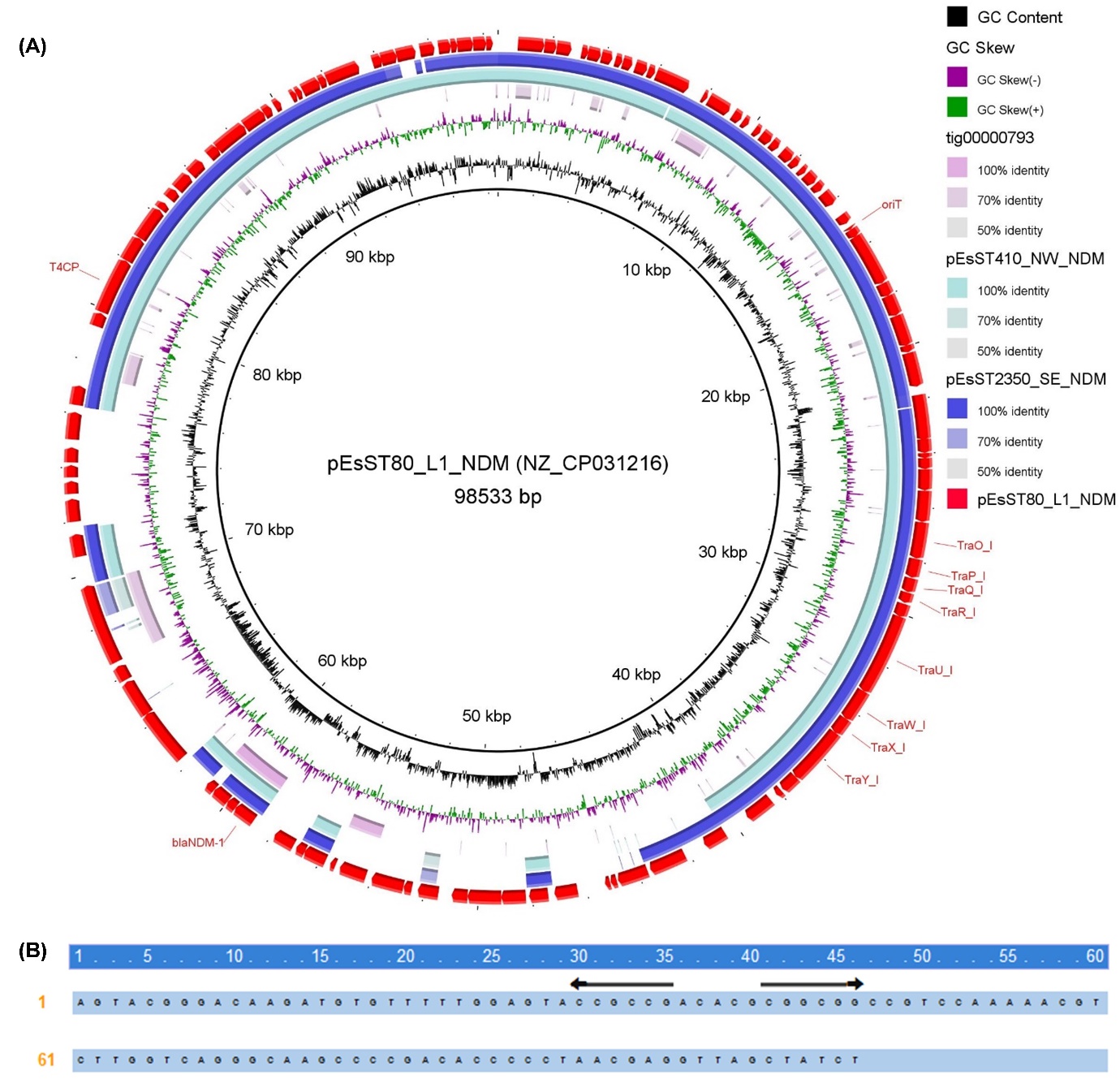


Figure S5


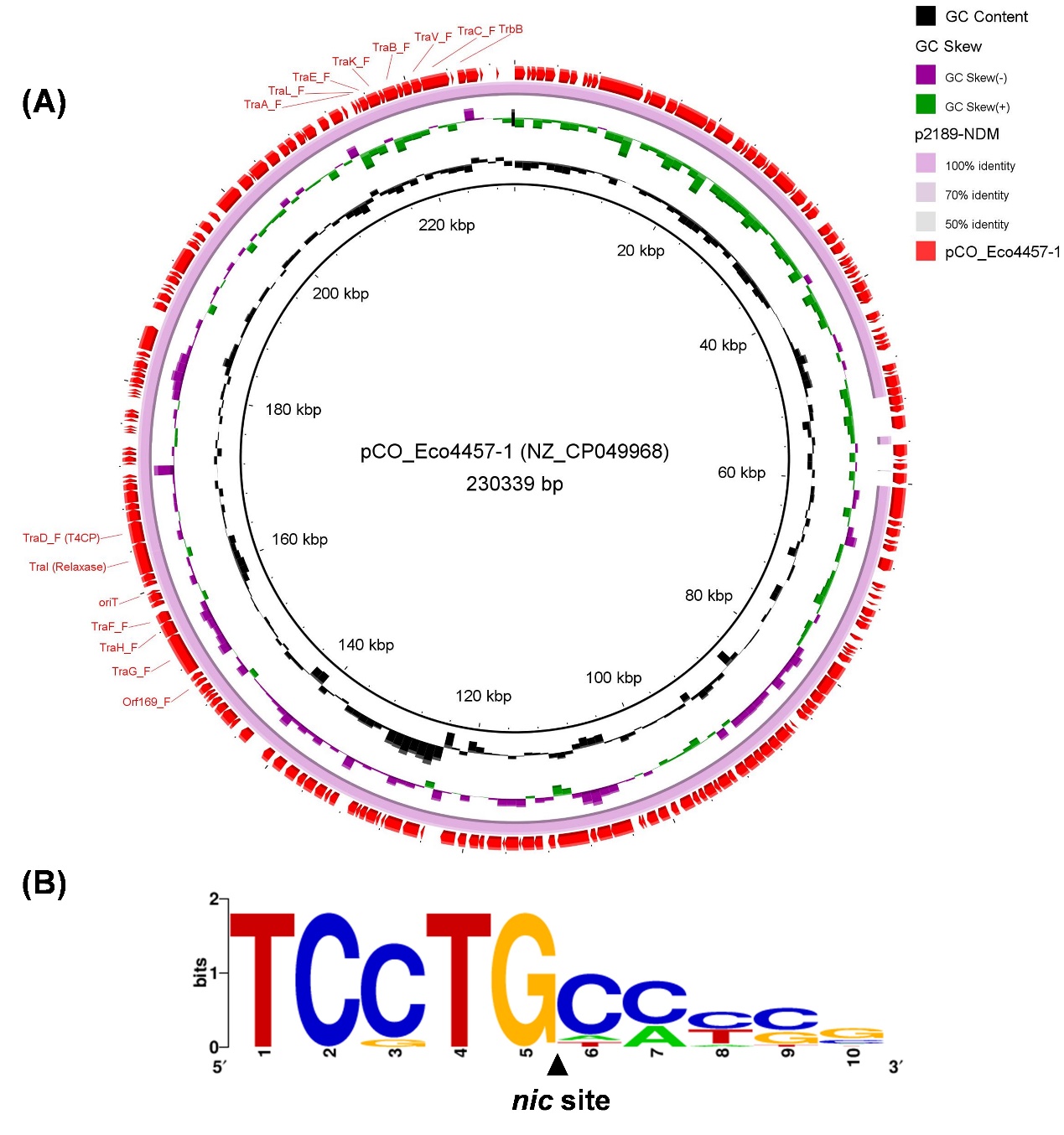


Figure S6
