# Supplementary figures and images for: Genetic Diversity and Characteristics of blaNDM-Positive Plasmids in Escherichia coli
Source: Front Microbiol. 2021 Nov 16;12:729952. doi: 10.3389/fmicb.2021.729952 (PMC8636099; doi:10.3389/fmicb.2021.729952)

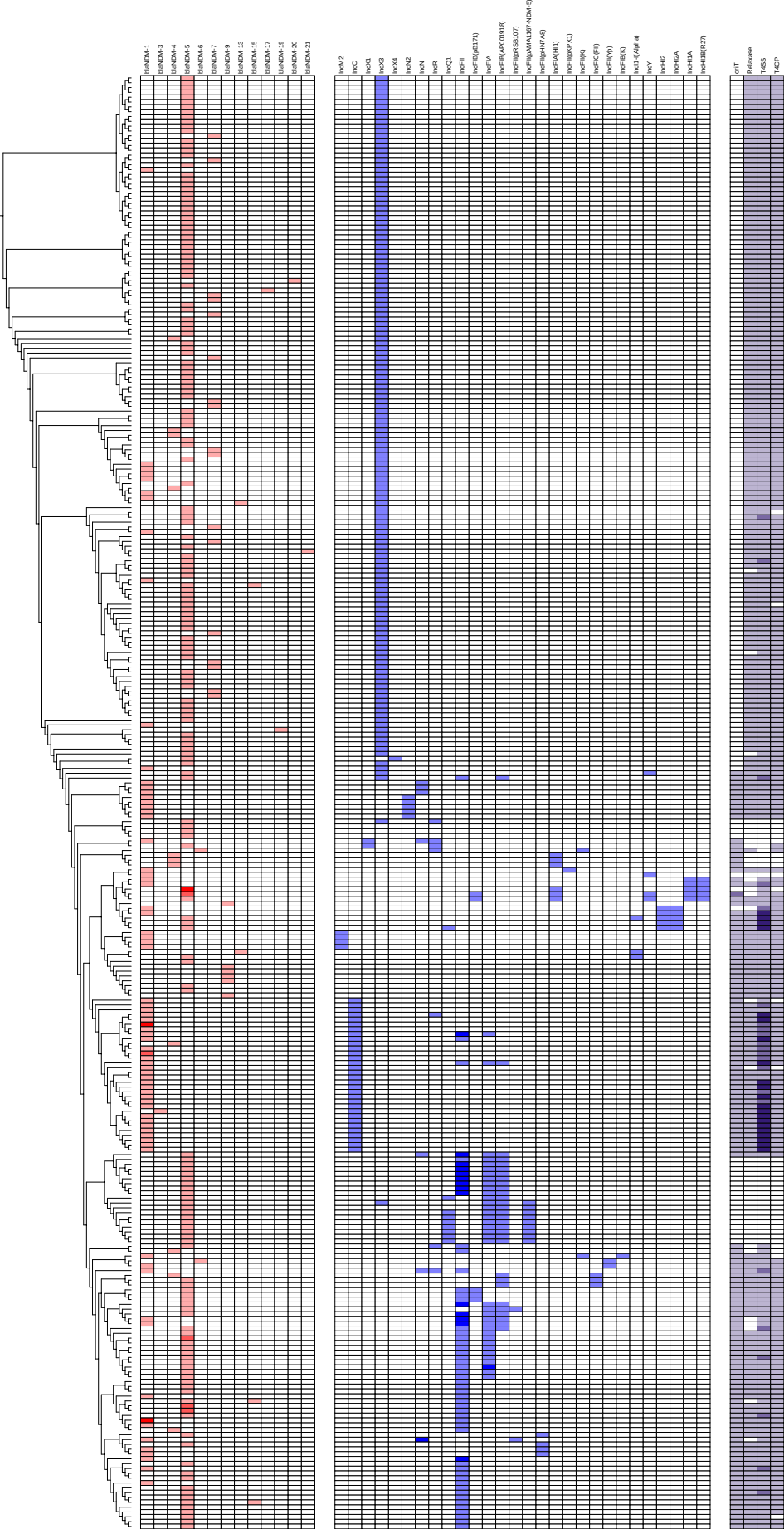

Supplement: Supplementary file 2 [file Data_Sheet_2.PDF]
